# Multi‐Omics Analysis Reveals Disturbances of Purine Metabolism and Glutamate Metabolism in the Hippocampus of Lipopolysaccharide‐Induced Mouse Model of Depression

**DOI:** 10.1002/brb3.70549

**Published:** 2025-05-11

**Authors:** Wen‐Wen Li, Rui Xiao, Xue‐Yi Chen, Jun‐Cai Pu, Jian‐Jun Chen, Hai‐Yang Wang, Lan‐Xiang Liu, Dan Li, Yang‐Dong Zhang, Wen‐Xia Li, Peng Xie

**Affiliations:** ^1^ Faculty of Basic Medicine, Department of Pathology Chongqing Medical University Chongqing China; ^2^ NHC Key Laboratory of Diagnosis and Treatment on Brain Functional Diseases The First Affiliated Hospital of Chongqing Medical University Chongqing China; ^3^ Molecular Medicine Diagnostic and Testing Center Chongqing Medical University Chongqing China; ^4^ Department of Pathology the First Affiliated Hospital of Chongqing Medical University Chongqing China; ^5^ Institute of Life Sciences Chongqing Medical University Chongqing China; ^6^ Chongqing Key Laboratory of Oral Diseases and Biomedical Sciences Stomatological Hospital of Chongqing Medical University Chongqing China; ^7^ Department of Neurology Yongchuan Hospital of Chongqing Medical University Chongqing China; ^8^ Department of Neurology The First Affiliated Hospital of Chongqing Medical University Chongqing China

**Keywords:** depression, proteomics, metabolomics, multi‐omics analysis

## Abstract

**Background:**

Depression is a global health concern characterized by high incidence, disability, and disease burden. Neuroimmunity, through the secretion of inflammatory mediators and mediation of neuroinflammation, plays a significant role in depression's pathogenesis. However, the underlying molecular mechanisms remain poorly understood.

**Methods:**

In this pioneering study, we employed a comprehensive multi‐omics approach, integrating 2‐DE proteomics, liquid chromatography mass spectrometry‐based metabolomics, and real‐time polymerase chain reaction (PCR) array, to investigate the hippocampal molecular profiles of lipopolysaccharide (LPS)‐induced immune inflammation‐related depression. This innovative approach aimed to explore the potential pathogenesis of depression by systematically integrating data across multiple molecular layers.

**Results:**

Compared to the control group, we identified 81 differential proteins, 44 differential metabolites, and 4 differential mRNAs in LPS‐treated mice. Integrated analysis of these multidimensional data revealed that purine metabolism and glutamate metabolism are the most significantly altered molecular pathways in LPS‐induced depression. Additionally, we constructed the corresponding compound‐reaction‐enzyme‐gene regulatory network.

**Conclusion:**

This study suggests that purine metabolism and glutamate metabolism may be the underlying mechanisms by which neuroinflammation regulates depression‐like behaviors. Our findings confirm the important role of immune inflammation in depression and provide a new clue for the diagnosis and treatment of this disorder. Notably, the multi‐omics approach employed in this study represents a pioneering effort in the field, providing unprecedented insights into the molecular mechanisms underlying depression.

## Graphical abstract

The study focused on a mouse model of depression induced by lipopolysaccharides (LPS) and employed innovative multi‐omics approaches, including two‐dimensional gel electrophoresis (2‐DE) for proteomics, liquid chromatography–mass spectrometry (LC–MS) for metabolomics, and real‐time polymerase chain reaction (PCR) microarray technology to analyze the hippocampus of mice. Through these techniques, we found that compared to the control group, there were 81 differentially expressed proteins, 44 differential metabolites, and 4 differential mRNAs in the mice treated with LPS. The integrated analysis of this multidimensional data revealed that purine metabolism and glutamate metabolism are the most significantly altered molecular pathways in depression induced by LPS.

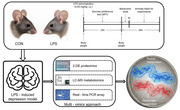

## 1 Introduction

Depressive disorder, characterized by pleasure loss, decreased interest, and energy deficiency, is common with high incidence and significant disease burden (Kendler 2020; McCarron et al. 2021; Park and Zarate 2019). Its pathogenesis remains incompletely understood, but inflammatory response is closely linked to depression (Beurel et al. 2020). Both patients and animal models show elevated pro‐inflammatory cytokines (Köhler et al. 2017; Menard et al. 2017), reduced by antidepressant treatment (Dionisie et al. 2021). Anti‐inflammatory drugs alleviate depressive symptoms, confirming the link (Kohler‐Forsberg et al. 2019). However, the underlying molecular mechanisms of this relationship are still not fully studied.

Lipopolysaccharide (LPS), produced by Gram‐negative bacteria, is crucial for bacterial outer membrane integrity and host–pathogen interactions (Whitfield and Trent 2014). LPS‐induced animal models show depression with hippocampal neuroinflammatory activation (Wei et al. 2021; Zhang et al. 2019; Zhao et al. 2019). LPS‐induced models consistently show increased immobility in the forced swim test and tail suspension test as well as reduced sucrose preference, indicating anhedonia. These behavioral changes are accompanied by elevated levels of pro‐inflammatory cytokines, such as IL‐1β, TNF‐α, and IL‐6, in the brain and periphery (Peng et al. 2012). Antidepressants alleviate LPS‐induced depression and inflammation (Li et al. 2021; Taniguti et al. 2019). Although these models adequately capture the immediate behavioral impacts of inflammation, they may not entirely mirror the chronic neuroinflammatory processes typically observed in human depression. Chronic depression frequently entails persistent, low‐grade inflammation, which could exhibit distinct molecular and behavioral manifestations compared to the acute inflammation induced by LPS (Zalli et al. 2016). Nevertheless, comprehensive systematic studies examining the full spectrum of molecular alterations in LPS‐induced inflammatory depression remain scarce.

Proteomics, metabolomics, and transcriptomics are high‐throughput assays detecting tens of thousands of proteins, metabolites, and genes simultaneously. These technologies study molecular regulation holistically and are crucial for understanding molecular alterations in diseases (Punzi et al. 2022; Rotello and Veenstra 2021; Schrimpe‐Rutledge et al. 2016). On this basis, it has been widely used to explore the mechanisms of heart diseases, diabetes, and brain diseases by integrating multi‐omics analysis (Guo et al. 2022; Song et al. 2021; Tsurumaki et al. 2015). Previous studies have explored the alterations in hippocampal metabolomic profiles resulting from LPS intervention. For example, Geng et al. reported that LPS intervention could lead to alterations in lipid metabolites in the hippocampus of rats (Geng, Guo, et al. 2020; Geng, Hao, et al. 2020). Li et al. (2019) found that LPS intervention could lead to disturbance of amino acid metabolism in the hippocampus of rats. One study integrating metabolomics and transcriptomics proposed the importance of phospholipid and tryptophan metabolism in LPS‐ and stress‐induced depression (Wang et al. 2021). However, there is a lack of multi‐omics studies to systematically explore the molecular alterations in the hippocampus after LPS intervention.

In our study, we established an LPS‐induced depression model and evaluated behavioral effects to investigate the impact of acute inflammation on depressive‐like behaviors. Utilizing a multi‐omics approach, we conducted a comprehensive analysis of proteomic, metabolomic, and polymerase chain reaction (PCR) microarray data, which elucidated the intricate connection between these behavioral changes and the underlying molecular alterations. This innovative strategy represents a pioneering effort to explore the mechanisms of LPS‐induced depression, providing both experimental and theoretical support for understanding the role of inflammation in this disorder. By integrating data from multiple molecular layers, our approach transcends previous work that focused solely on individual molecular components, enabling a systematic exploration of the integrated molecular landscape in the hippocampus following LPS treatment.

## 2 Materials and Methods

### 2.1 Animals and Treatments

Male CD‐1 mice (10–14 weeks old, 35–40 g) were supplied by experimental animal center of Chongqing Medical University. All mice were housed in single cages, under standard environmental conditions (23°C ± 1°C, 12‐h light–dark cycle, humidity 50% ± 5%), and all had access to food and water ad libitum. After 10 days of adaptation, 40 CD‐1 mice were randomly divided into 2 groups (*n* = 20/group): (1) control group (CON group); (2) LPS‐induced depressive‐like behavior (LPS group). In the LPS and CON groups, sterile LPS (0.83 mg/kg) or sterile saline (1 mL/kg) was injected intraperitoneally, respectively. The injection dose of LPS was based on our previous study (Wu et al. 2016).

To ensure the statistical power of our study, sample size calculations were performed using GPower software. For the behavioral tests and overall study design, we aimed to detect significant differences in depressive‐like behaviors between the LPS‐treated and control groups. Assuming an effect size (d) of 1.0, an α error probability of 0.05, and a power (1‐β error probability) of 0.80, GPower calculations indicated that a minimum of 17 samples per group were required to detect significant differences in behavioral measures. To account for potential dropouts and ensure robust statistical analysis, we chose to use 20 samples per group, resulting in a total of 40 mice.

This research was conducted in accordance with recommendations of the Guide for the Care and Use of Laboratory Animals. The experimental protocol of this study, which involved the use of 40 animals, was approved by the ethics committee of Chongqing Medical University (approval no.: IACUC‐CQMU‐2010‐0031) (He et al. 2020).

The 24‐h post‐LPS timeline was chosen on the basis of previous studies demonstrating significant behavioral and molecular changes within this timeframe, reflecting the acute phase of neuroinflammation (Köhler et al. 2017; Taniguti et al. 2019). On the basis of previous studies and relevant literature, the period of 24–48 h after injection is a critical window for observing the effects of LPS on animal behavior (Deyama et al. 2025). Therefore, we chose to conduct behavioral testing within 28 h after injection (O'Connor et al. 2009). Acute inflammation models are valuable for understanding the immediate molecular and behavioral alterations associated with neuroinflammation, which are critical for elucidating the underlying mechanisms of depression. However, it is acknowledged that chronic exposure to inflammatory stimuli may result in different molecular and behavioral profiles, which are not fully captured in acute models (Carstensen et al. 2019; Irwin 2011).

### 2.2 Behavioral Testing

As shown in Figure S1, behavioral tests were performed within 28 h after injection to comprehensively assess the immediate and short‐term impacts of inflammation on depressive‐like behaviors. Body weight (BW) was recorded before and 24 h after LPS treatment to monitor any acute changes indicative of overall health status. The sucrose preference test was performed within 24 h after intervention to assess anhedonia core symptom of depression characterized by reduced pleasure‐seeking behavior (Primo et al. 2023; Willner 1984). Briefly, after LPS injection, each mouse was given one bottle of 2% sucrose water and one bottle of pure water at the same time, and the consumption of sucrose water and pure water (g) during 24 h was recorded. Sucrose preference was calculated as the percentage of the consumed sucrose water relative to the total amount of liquid intake. A significant reduction in sucrose preference indicates anhedonia, suggesting that LPS‐induced inflammation affects reward‐related behaviors.

The open field test was performed to assess the motor ability and general anxiety‐like behavior of both groups. Mice were individually placed in a clean 44.5 cm × 44.5 cm × 45 cm chamber for free movement. The total distance traveled in 5 min was recorded using the SMART video‐tracking system software (Panlab, Spain). This test helps to determine whether observed behavioral changes are specific to depressive‐like symptoms rather than general motor dysfunction or anxiety.

The forced swim test was used to investigate depression‐like behaviors, a key component of depressive‐like behaviors. Mice were individually placed in a Plexiglas cylinder (15 cm in diameter and 30 cm in height, with a water depth of 15 cm and a water temperature of 23°C ± 1°C). The immobility time during 5 min was analyzed using the SMART video‐tracking system software. Immobility time reflects the duration during which the mouse remains motionless, indicating a state of depression‐like behaviors. A significant increase in immobility time suggests that LPS‐induced inflammation leads to increased depression‐like behaviors. Although the FST is widely used and provides valuable insights into depressive‐like behaviors, it has several limitations. The test may not fully capture the complexity of human depression and can be influenced by factors such as strain differences, prior handling, and environmental conditions. Additionally, the FST has been criticized for its potential to measure nonspecific stress responses rather than specific depressive‐like behaviors. Despite these limitations, the FST remains a key assay for evaluating depression‐like behaviors in acute inflammation models.

The tail suspension test was also used to assess depression‐like behaviors. A small piece of tape was wrapped around the tail of each mouse, and then mice were suspended individually from a hook. The immobility time during 5 min was analyzed using the SMART video‐tracking system software. Similar to the FST, increased immobility time in the TST indicates depression‐like behaviors and supports the hypothesis that LPS‐induced inflammation leads to significant depressive‐like behaviors.

### 2.3 Metabolite Analysis in Hippocampal Region Based On UPLC‐Q/TOF‐MS Technique

Once the behavioral experiments were completed, mice were sacrificed by dislocation of the cervical vertebrae. Then hippocampus tissues were rapidly removed from the brain and transferred to −80°C for storage.

For metabolomics analysis, eight hippocampus tissues in each group were selected for metabolomic assay. For each mouse, 50 mg of the frozen tissue samples was quickly weighed and added to 1 mL of ice‐cold chloroform/methanol/water, and then tissues were homogenized on ice. The supernatant was removed by centrifugation at 4°C for 14,000 *g* × 15 min. Acquity Ultra Performance LC Q‐TOF premier (Waters) equipped with a Waters ASQUITY UPLC C18 column (100 mm × 2.1 mm, 1.7 µm) and a UPLC C18 guard column (2.1 mm × 5 mm) was used for metabolomics analysis. Chromatographic separation conditions were as follows: column temperature, 40°C; flow rate, 0.4 mL/min. Mobile phases included water containing 0.1% formic acid (A) and acetonitrile + 0.1% formic acid (B). The gradient elution procedure was as follows: 0–3 min, 99% A; 3–5 min, 80% A; 5–12 min, 40% A; 12–14.5 min, 0% A.

The sample was analyzed by UPLC‐Q/TOF‐MS in positive mode and negative mode for metabolite identification. The positive ion mode conditions were set as follows: capillary voltage, 2.5 kV; sampling cone, 35 kV; source temperature, 100°C; desolvation gas temperature, 300°C; cone gas flow, 50 L/h; desolvation gas flow, 700 L/h; and extraction cone, 4 V. The negative ion mode conditions were set as follows: capillary voltage, 2.5 kV; sampling cone, 50 kV; source temperature, 100°C; desolvation temperature, 300°C; cone gas flow, 50 L/h; desolvation gas flow, 700 L/h; extraction cone, 4 V. In the positive and negative ion modes, scan time was set as 0.03 s, inter scan time was set as 0.02 s, and data acquisition range was set as 50–1000 *m/z*.

Raw liquid chromatography–mass spectrometry (LC–MS) data underwent initial processing utilizing proprietary software crafted on the R platform. This process encompassed peak detection, integration, and alignment across all samples to compile a comprehensive data matrix. To address any drifts in the chromatographic system, retention time correction was implemented, ensuring consistent alignment of peaks corresponding to identical metabolites across various runs.

To augment data quality, noise reduction techniques were employed to diminish background interference. Missing values, potentially stemming from low signal intensity or instrument detection thresholds, were imputed using probabilistic models, thereby guaranteeing a complete data matrix for subsequent analysis. Normalization was executed using the total ion current (TIC) method, which scaled the sum of all ion intensities in each sample to a constant value. This approach corrected for variations in sample injection volume, ionization efficiency, and other technical factors impacting overall ion intensity.

Furthermore, data were log‐transformed (log2) to stabilize variance and enhance the normality of the distribution, crucial for statistical analysis. To prevent metabolites with high abundance from dominating the analysis, data were scaled using unit variance scaling (UVS), which scaled each variable by its standard deviation, ensuring equal contribution from each metabolite. Additionally, data were mean‐centered by subtracting the mean value of each variable from the dataset, yielding a mean of zero for each variable. This step mitigated systematic biases and enhanced result interpretability.

Following preprocessing, the data matrices were imported into SIMCA‐P software (version 11.0) for principal component analysis (PCA) and partial least squares discriminant analysis (PLS‐DA). Differential metabolites were identified on the basis of Variable Importance in the Projection (VIP) values exceeding 1 and a Student's *t*‐test *p* value below 0.05.

To further elucidate the biological functions of these differential metabolites, pathway analysis was performed using MetaboAnalyst 5.0 (Pang et al. 2021). The Human Metabolome Database (HMDB) IDs of the differential metabolites were uploaded to MetaboAnalyst, the hypergeometric test was used to enrich the significantly altered pathways, and “Mus musculus (Kyoto Encyclopedia of Genes and Genomes [KEGG])” was used as the enriched pathway library. Pathways with false discovery rate (FDR) < 0.05 were defined as significantly altered pathways.

### 2.4 Proteomics Analysis in Hippocampal Region Based On 2‐DE Technique

Ten hippocampal samples were selected from each group for bidirectional electrophoresis (2‐DE) experiments. The proteomics analysis was similar to our previous study (Tian et al. 2020). Briefly, proteins were extracted using an acetone solution of 10% TCA with 0.2% DTT, dried, and dissolved in rehydration solution (7 M urea, 2 M thiourea, 4% CHAPS, 50 mM DTT, and 0.2% bio‐lyte). The concentrations of proteins were assayed by the Bradford method. An amount of 200 µg of protein was added to 17 cm long gel strips for duplex electrophoresis. Every gel was run in triplicate. After silver staining, the strips were scanned and analyzed using PD‐Quest Advanced two‐way electrophoresis gel image analysis software (V. 8.0.1, Bio‐Rad, USA). Protein spots with fold changes greater than 1.5 were considered to be statistically significant between the two groups. A 4800 Plus MALDI TOF/TOF analyzer (ABI, USA) was used to identify differential proteins of the protein spots. The spectra were searched against the UniProt‐Mus_musculus (144127).

To further elucidate the biological functions of these differential proteins, we performed Gene Ontology (GO) analysis and KEGG pathway analysis using clusterProfiler (Yu et al. 2012). GO analysis can identify the significantly altered biological process (BP), cellular component (CC), and molecular function (MF) terms associated with the differential proteins. GO terms with FDR < 0.05 were selected as significantly altered terms. For KEGG pathway analysis, “org.Mm.eg.db” was selected as the backend library, and pathways with FDR < 0.05 were selected as significantly altered pathways.

### 2.5 Real‐Time PCR Array

A SuperArray Bioscience SYBR Green real‐time fluorescent quantitative PCR chip (CAPM11401) was used for high‐throughput detection of altered genes in the hippocampus of LPS and CON mice. Details of the PCR Array were described in a previous study (Arikawa et al. 2008). Briefly, six hippocampi were selected from each group and tested individually. An amount of 50 mg of hippocampal samples was weighted, and RNA was extracted using the RNeasy MinElute Cleanup Kit, according to the instructions. RNA concentration and purity were measured using NanoDrop ND‐1000. SuperScript III Reverse Transcriptase (Invitrogen) was used for cDNA synthesis, PCR master mix (SuperArray) was used for sample preparation, and 7900HT 96‐well block from ABI was used for PCR experiments. The ΔΔCt method was used to calculate the expression of each gene.

### 2.6 Joint Pathway Analysis

To integrate differential proteins, metabolites, and transcription molecules, we used joint pathway analysis. Gene symbols and HMDB IDs of differential molecules were uploaded to MetaboAnalyst for hypergeometric test enrichment, using “All pathways (integrated)” as the library. Pathways with FDR < 0.05 were considered significant. To further validate these findings and deepen our understanding of the data, we subsequently employed Ingenuity Pathway Analysis (IPA) to perform additional pathway analysis on the integrated data. This step not only helped us confirm the results obtained from the joint pathway analysis but also provided in‐depth insights into the potential roles of differential molecules in BPs. Furthermore, to explore gene‐metabolite interactions, we constructed metabolite‐protein‐gene networks in Cytoscape 3.9.0 using MetScape (Gao et al. 2010). Cytoscape's plug‐in molecular complex detection technology (MCODE) was used to analyze key functional modules. Set the selection criteria as K‐core = 2, degree cutoff = 2, max depth = 100, and node score cutoff = 0.2.

### 2.7 Statistical Analysis

SPSS 21.0 software (IBM, USA) was used for behavioral data analysis. The behavioral results of the two groups of animals were compared using the independent sample Student's *t*‐test. *p* < 0.05 was considered statistically significant. Differential metabolites were identified using a combination of VIP values and Student's *t*‐tests. Metabolites with VIP values >1 and Student's *t*‐test *p* < 0.05 were considered significantly altered. Proteins were considered differentially expressed if their expression levels showed at least a 1.5‐fold increase or decrease between the LPS‐treated and control groups. This threshold was chosen to balance sensitivity and specificity in detecting significant changes while minimizing false positives.

## 3 Results

### 3.1 Effects of LPS on the Depressive‐Like Behaviors of Mice

**FIGURE 1:**
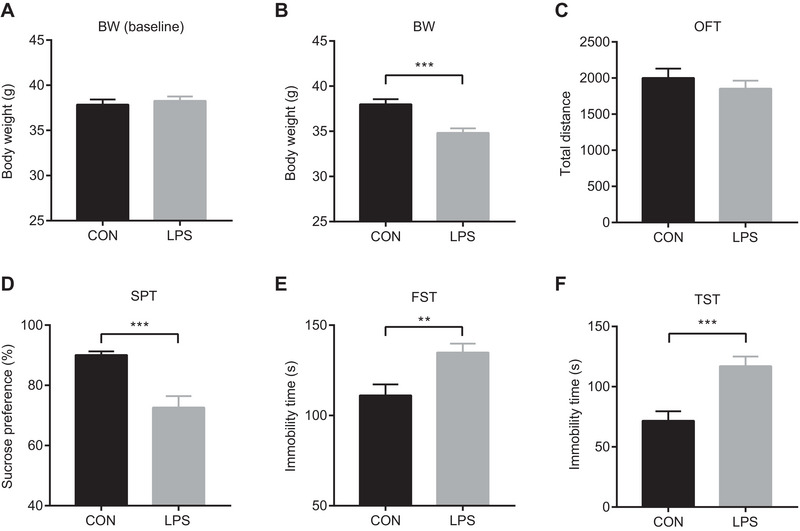
The change in lipopolysaccharide (LPS)‐induced depressive‐like behaviors. Mice were injected with saline or LPS (0.83 mg/kg). (A) Baseline body weight (BW) of mice before treatment with saline or LPS (0.83 mg/kg). No significant difference was observed between the control (CON) and LPS groups at baseline (*p* > 0.05), indicating that the initial body weight did not influence the subsequent behavioral assessments. (B) Body weight of mice 24 h after administration of LPS. The data were analyzed using an independent samples *t*‐test. A significant decrease in body weight was observed in the LPS group compared to the control group (***p < 0.001), suggesting that LPS administration may lead to altered energy metabolism. (C) Total locomotor activity of mice in the open field test (OFT) following LPS administration. Data were analyzed using an independent samples *t*‐test with SPSS 21.0 software. No significant difference in total distance traveled was found between the LPS and control groups (*p* > 0.05), indicating that LPS did not significantly affect the locomotor activity of the mice. (D) Sucrose preference percentage of mice in the sucrose preference test (SPT) following LPS administration. Data were analyzed using an independent samples *t*‐test. A significant decrease in sucrose preference was observed in the LPS group compared to the control group (***p < 0.001), indicating anhedonia, a core symptom of depression. (E) Immobility time of mice in the forced swim test (FST) following LPS administration. Data were analyzed using an independent samples t‐test with SPSS 21.0 software. A significant increase in immobility time was observed in the LPS group compared to the control group (**p < 0.01), suggesting that LPS administration may induce depression‐like behaviors, a key symptom of depression. (F) Immobility time of mice in the tail suspension test (TST) following LPS administration. Data were analyzed using an independent samples *t*‐test. A significant increase in immobility time was observed in the LPS group compared to the control group (***p < 0.001), further supporting the induction of depression‐like behaviors by LPS. Data were represented as mean and SEM. Bars indicate statistical differences between groups. **p < 0.01, ***p < 0.001.

There was no significant difference in BW between the two groups at baseline (*t* = −0.57, *p* = 0.572; Figure 1A). The mean BW for the control group was 37.8 g (SD = 2.5 g, 95%CI = 36.6–39.1 g), and for the LPS group, it was 38.3 g (SD = 2.1 g, 95%IC = 37.3–39.3 g). The effect size (Cohen's *d*) was 0.18. After 24 h of LPS intervention, BW was significantly decreased in the LPS group (*t* = 4.17, *p* < 0.001; Figure 1B). The mean BW for the control group was 38.0 g (SD = 2.5 g, 95%IC = 36.8–39.2 g), whereas for the LPS group, it was 34.8 g (SD = 2.2 g, 95%IC = 33.8–35.9 g). The effect size (Cohen's *d*) was 1.33.

The results of the open field test showed no significant differences in the total distance between both groups (*t* = 0.85, *p* = 0.399; Figure 1C). The mean distance traveled for the control group was 2000.3 cm (SD = 577.8 cm, 95%IC = 1729.9–2270.7 cm), and for the LPS group, it was 1854.1 cm (SD = 463.1 cm, 95%IC = 1623.8–2084.4 cm). The effect size (Cohen's *d*) was 0.28. This suggests that LPS intervention did not cause a significant change in the locomotor ability of the mice.

The results of the sucrose preference test showed that sucrose preference was significantly reduced in the LPS group (*t* = 4.41, *p* < 0.001; Figure 1D). The mean sucrose preference for the control group was 90.1% (SD = 5.1%, 95%IC = 87.7%–92.5%), whereas for the LPS group, it was 72.6% (SD = 17.0%, 95%IC = 64.7%–80.5%). The effect size (Cohen's *d*) was 1.40. This suggests that the mice exhibited pleasure‐deficit‐like behavior after the LPS intervention.

The FST results showed that the immobility time of mice in the LPS group was significantly prolonged (*t* = −3.06, *p* = 0.005, Figure 1E). The mean immobility time for the control group was 111.2 s (SD = 24.3 s, 95%IC = 98.2–124.1 s), whereas for the LPS group, it was 134.9 s (SD = 20.1 s, 95%IC = 124.5–145.2 s). The effect size (Cohen's *d*) was 1.06. This indicates that LPS intervention caused increased depression‐like behaviors in the animals.

The TST results showed that the immobility time of mice in the LPS group was significantly prolonged (*t* = −4.05, *p* < 0.001, Figure 1F). The mean immobility time for the control group was 71.7 s (SD = 31.5 s, 95%IC = 54.9–88.5 s), whereas for the LPS group, it was 117.1 s (SD = 30.9 s, 95%IC = 100.0–134.2 s). The effect size (Cohen's *d*) was 1.45. This further supports the finding that LPS intervention caused depression‐like behaviors.

Taken together, these results suggest that depression‐like behaviors, characterized by anhedonia and increased depression‐like behaviors, emerged in mice 24 h after LPS injection. The lack of significant changes in locomotor activity indicates that these behavioral changes are specific to depressive‐like symptoms rather than general motor dysfunction.

### 3.2 Effects of LPS on the Hippocampal Metabolomic Profiles of Mice

PCA analysis was performed on the data obtained in positive and negative modes.

**FIGURE 2:**
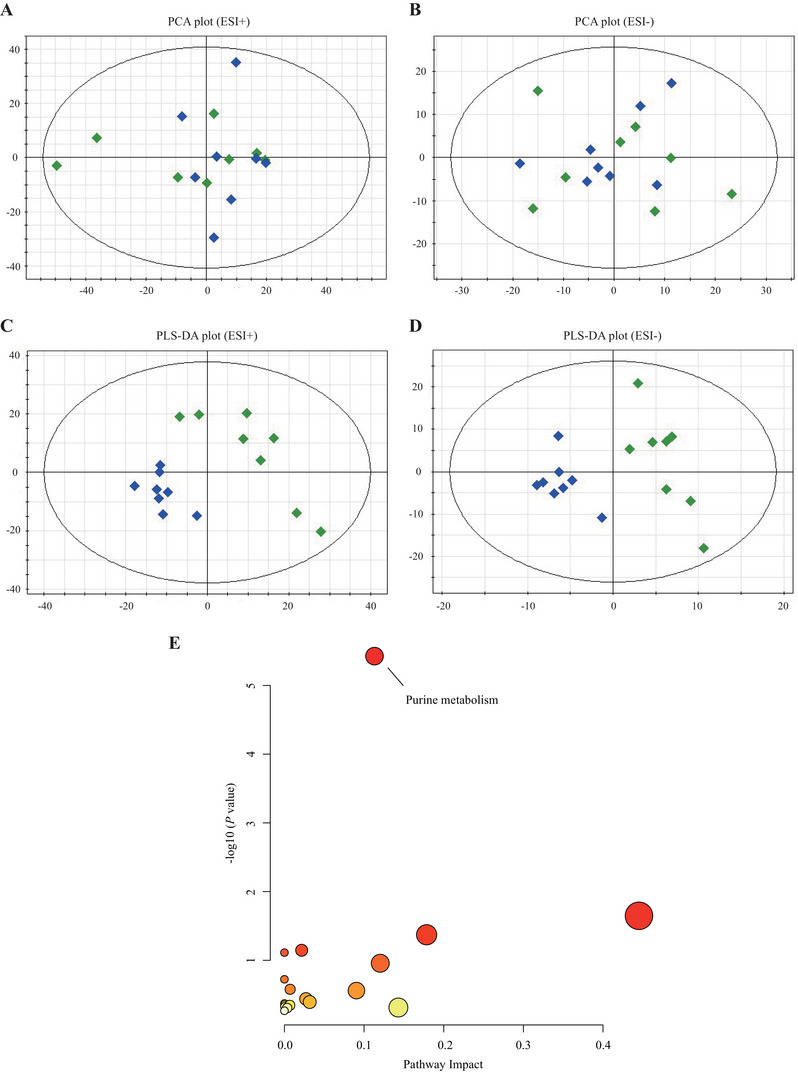
Metabolomic analysis of hippocampi from LPS and CON group mice. (A) Principal component analysis (PCA) plot in positive mode (ESI+) showing the distribution of hippocampal metabolomic profiles from LPS (blue) and control (CON, green) group mice. PCA was performed using the SIMCA‐P software to reduce dimensionality and visualize differences between groups. Each point represents a single mouse, and the distance between points indicates the degree of similarity in metabolite profiles. The PCA score explains the variance captured by each principal component, with PC1 and PC2 accounting for the majority of the metabolic differences observed. (B) PCA plot in negative mode (ESI−) illustrating the metabolomic profiles of the LPS and control groups. This analysis helps in identifying the metabolic signatures associated with LPS treatment. The PCA model was validated using *Q* ^2^ and scree plots to assess the appropriate number of components to retain. (C) Partial least squares discriminant analysis (PLS‐DA) plot in positive mode (ESI+) differentiating LPS (blue) and control (CON, green) groups. PLS‐DA maximizes the separation between groups while minimizing the variation within each group. The VIP (variable importance in projection) scores and *p* values indicate the contribution and statistical significance of each metabolite to the group discrimination. (D) PLS‐DA plot in negative mode (ESI−) showing the metabolic profiles of LPS and control groups. This analysis further supports the PCA findings by providing a more detailed view of the metabolic changes associated with LPS treatment. The significant metabolites identified by PLS‐DA are potential biomarkers of the LPS‐induced depressive‐like behavior. (E) Significantly enriched pathways of the significantly altered proteins. Pathway analysis was conducted using MetaboAnalyst 5.0 to identify the biological processes and pathways impacted by LPS treatment. Pathways with an FDR (false discovery rate) <0.05 were considered significantly enriched, indicating a high likelihood of being associated with the observed metabolic changes. The name of significantly enriched pathway (FDR < 0.05) is shown.

Two principal components were obtained in positive mode (R2X = 0.403 and Q2 = 0.071; Figure 2A), and three principal components were obtained in negative mode (R2X = 0.546 and Q2 = 0.111; Figure 2B). The PCA results show that the statistical models were reliable. Differential metabolites in the positive and negative modes were screened by PLS‐DA (Figure 2C,D), respectively. The results showed that 26 metabolites were obtained in positive mode and 19 differential metabolites in negative mode. Oxidized glutathione was identified as differential metabolites in both modes. Details of these differential metabolites are presented in Table 1.

**TABLE 1:** Differential metabolites of lipopolysaccharide (LPS) and control (CON) group mice.

Mode	Metabolite	HMDB ID	*m/z*	rt	VIP	*p* value	Fold change (LPS/Dep)	Biological context
ESI+	Choline	HMDB0000097	104.1066	0.67	2.53	0.001	0.94	Glycerophospholipid metabolism, cell membrane; essential for the synthesis of phospholipids and sphingomyelin, critical for maintaining cell membrane structure and function
	Oxidized glutathione	HMDB0003337	613.1589	0.71	2.16	0.006	1.11	Glutathione metabolism, cytoplasm; acts as an antioxidant by scavenging reactive oxygen species and protecting cells from oxidative stress
	Deamino nicotinamide adenine dinucleotide	NA	665.1114	0.74	2.18	0.006	1.17	NAD metabolism, Mitochondrion; important cofactor in redox reactions, particularly in energy metabolism
	Cyclic ADP‐Ribose	HMDB0249529	542.0568	0.76	2.04	0.011	1.11	Calcium signaling pathway, cytoplasm; functions as a second messenger in calcium signaling, regulating calcium ion release from intracellular stores
	Acetylcarnitine	HMDB0000201	204.1224	0.77	2.59	<0.001	1.09	Fatty acid beta‐oxidation, mitochondrion; transports fatty acids into the mitochondria for beta‐oxidation
	l‐Tryptophan	HMDB0000929	205.0962	0.77	1.67	0.046	1.05	Tryptophan metabolism, cytoplasm; precursor for serotonin and melatonin synthesis, important for mood regulation and sleep
	Guanosine monophosphate	HMDB0001397	364.0598	0.77	1.80	0.029	0.93	Purine metabolism, cytoplasm/nucleus; involved in nucleotide synthesis and signaling pathways
	Hypoxanthine	HMDB0000157	137.0492	1.25	2.37	0.002	0.82	Purine metabolism, cytoplasm; intermediate in purine catabolism, can be converted to uric acid
	N6‐(1,2‐dicarboxyethyl)‐AMP	HMDB0000536	464.082	1.30	2.37	0.002	0.78	Purine metabolism, cytoplasm; modified form of AMP, may play a role in energy metabolism
	Pantothenic acid	HMDB0000210	220.1186	1.52	2.58	<0.001	1.18	Coenzyme A biosynthesis, cytoplasm; precursor for coenzyme A (CoA), essential for fatty acid synthesis and energy metabolism
	Butyryl‐l‐carnitine	HMDB0002013	232.1503	1.65	1.88	0.022	1.63	Fatty acid beta‐oxidation, mitochondrion; transports butyryl‐CoA into the mitochondria for beta‐oxidation
	2‐Amino hexadecanoic acid	NA	272.2588	4.58	2.50	0.001	1.18	Fatty acid metabolism, membrane; long‐chain fatty acid derivative, may play a role in membrane lipid composition
	Sphinganine	HMDB0000269	302.3054	5.17	2.31	0.003	1.31	Sphingolipid metabolism, membrane; precursor for sphingosine and sphingomyelin, important for maintaining membrane integrity
	Sphingosine‐1‐phosphate	HMDB0000277	380.255	5.33	1.78	0.032	1.10	Sphingolipid metabolism, membrane; signaling molecule involved in cell proliferation, migration, and survival
	*N*‐Arachidonoyl‐3‐hydroxy‐γ‐aminobutyric acid	NA	406.2923	5.63	1.95	0.016	1.23	Endocannabinoid signaling, membrane; fatty acid amide derivative, may play a role in pain perception and inflammation
	3‐Methoxy PGF1α	NA	387.285	5.69	1.89	0.021	1.22	Prostaglandin synthesis and regulation, cytoplasm; prostaglandin derivative involved in inflammation and pain regulation
	Linoleyl carnitine	HMDB0006469	424.3402	5.84	2.41	0.001	1.22	Fatty acid beta‐oxidation, mitochondrion; transports linoleyl‐CoA into the mitochondria for beta‐oxidation
	25‐Hydroxyvitamin D3	HMDB0003550	401.3453	6.15	1.68	0.044	1.10	Vitamin D metabolism, cytoplasm/nucleus; active form of vitamin D, involved in calcium and phosphorus homeostasis
	Palmitoyl‐l‐carnitine	HMDB0000222	400.3422	6.15	1.66	0.048	1.11	Fatty acid beta‐oxidation, mitochondrion; transports palmitoyl‐CoA into the mitochondria for beta‐oxidation
	LysoPE (24:6)	HMDB0011499	554.3142	6.15	1.68	0.045	1.19	Glycerophospholipid metabolism, cell membrane; phospholipid derivative, may play a role in membrane structure and function
	PC (19:0)	NA	532.3338	6.33	1.86	0.023	1.30	Glycerophospholipid metabolism, cell membrane; phosphatidylcholine derivative, major component of cell membranes
	PGF2α alcohol methyl ether	NA	355.2827	7.43	1.72	0.038	1.45	Prostaglandin synthesis and regulation, cytoplasm; modified prostaglandin derivative, may have altered biological activity
	3‐Hexanoyl‐NBD cholesterol	NA	663.4567	10.42	1.85	0.024	0.75	Lipid metabolism, membrane; involved in cholesterol transport and membrane fluidity
	PC (35:5)	HMDB0007951	766.53	10.53	1.66	0.048	0.69	Glycerophospholipid metabolism, membrane; structural component of cell membranes, signal transduction
	PC (34:1)	HMDB0007879	760.5841	11.90	1.66	0.047	0.84	Glycerophospholipid metabolism, membrane; structural component of cell membranes, signal transduction
	PC (36:4)	HMDB0007889	798.5452	11.95	1.84	0.026	0.79	Glycerophospholipid metabolism, membrane; structural component of cell membranes, signal transduction
ESI−	Oxidized glutathione	HMDB0003337	611.145	0.59	2.32	0.005	1.10	Glutathione metabolism, cytoplasm; acts as an antioxidant by scavenging reactive oxygen species and protecting cells from oxidative stress
	CMP‐*N*‐acetylneuraminic acid	HMDB0001176	613.1423	0.60	2.21	0.009	1.06	*N*‐Glycan biosynthesis; amino sugar and nucleotide sugar metabolism, nucleus, cytoplasm; precursor for sialic acid synthesis, involved in glycoprotein and glycolipid biosynthesis
	Malic acid	HMDB0000744	133.0106	0.62	2.26	0.007	0.90	Citrate cycle (TCA cycle), mitochondria; intermediate in energy production, pH regulation
	Citric acid	HMDB0000094	191.015	0.63	2.22	0.008	0.93	Citrate cycle (TCA cycle), mitochondria; initiates TCA cycle, essential for ATP production
	ADP‐ribose	HMDB0001178	558.0609	0.66	2.41	0.003	0.78	NAD+ metabolism, cytoplasm, nucleus; precursor for NAD+ synthesis, energy transfer
	Inosine diphosphate	HMDB0003335	427.0125	0.67	2.25	0.007	1.10	Purine metabolism, cytoplasm, nucleus; precursor for ATP and GTP synthesis, signal transduction
	ADP	HMDB0001341	426.0205	0.68	2.53	0.002	1.12	Purine metabolism; pyrimidine metabolism, cytoplasm; energy currency, precursor for ATP synthesis
	Adenosine triphosphate	HMDB0000538	505.9784	0.68	1.88	0.033	1.11	Energy metabolism, cytoplasm, mitochondria; primary energy currency, drives various cellular processes
	Deamino‐NAD+	HMDB0001179	664.0958	0.69	2.79	<0.001	1.19	NAD+ metabolism, cytoplasm, nucleus; derivative of NAD+, potentially involved in redox reactions
	Nicotinamide adenine dinucleotide	HMDB0000902	662.0828	0.70	2.79	<0.001	1.16	NAD+ metabolism, cytoplasm, mitochondria, nucleus; coenzyme in energy metabolism, essential for glycolysis and TCA cycle
	Inosine	HMDB0000195	267.075	1.19	2.11	0.014	0.85	Purine metabolism, cytoplasm; energy source, signaling molecule in immune responses
	*N*‐(3‐Oxododecanoyl) homoserine lactone	NA	296.1792	2.75	2.10	0.014	4.61	Quorum sensing in bacteria, extracellular; signal molecule for bacterial communication and behavior regulation
	*N*‐Palmitoyl taurine	HMDB0240594	362.2326	2.76	1.88	0.032	0.90	Lipid metabolism, membrane; membrane stabilizer, osmoregulation
	*cis*‐7‐Hexadecenoic Acid	HMDB0002186	253.2154	5.32	1.79	0.044	0.92	Fatty acid metabolism, membrane; component of phospholipids, energy storage
	PG (44:4)	NA	881.6341	11.49	1.95	0.025	1.62	Glycerophospholipid metabolism, membrane; structural component of cell membranes, signal transduction
	PC (42:5)	HMDB0008257	862.6109	11.49	1.99	0.022	1.32	Glycerophospholipid metabolism, membrane; structural component of cell membranes, signal transduction
	PI (39:1)	NA	905.6277	11.49	1.84	0.038	1.30	Glycerophospholipid metabolism; inositol phosphate metabolism, membrane; structural component of cell membranes, signal transduction, cell signaling
	PG (44:5)	NA	879.6107	11.50	2.11	0.014	1.58	Glycerophospholipid metabolism, membrane; structural component of cell membranes, signal transduction
	PG (43:6)	NA	880.6192	11.50	2.05	0.017	1.56	Glycerophospholipid metabolism, membrane; structural component of cell membranes, signal transduction
	

*Note*: Metabolites with fold changes >1 and <1 are significantly increased and decreased, respectively.

Abbreviations: HMDB, Human Metabolome Database; NA, not available.

We then used pathway analysis to explore the biological functions of these differential metabolites. Pathway analysis identified three nominal differential pathways, including purine metabolism, nicotinate and nicotinamide metabolism, and sphingolipid metabolism. After FDR correction, purine metabolism was the only significantly enriched pathway (FDR < 0.001; Figure 2E).

### 3.3 Effects of LPS on the Hippocampal Protein Expressions of Mice

**FIGURE 3:**
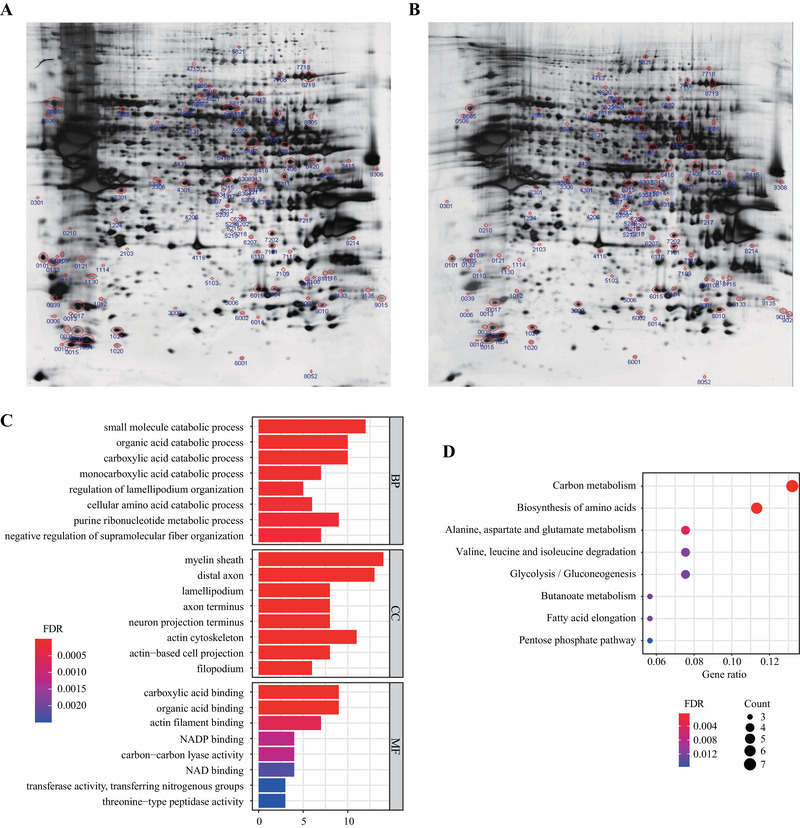
Proteomics analysis of hippocampi from LPS and CON group mice. (A) 2‐DE map of hippocampal proteins from the lipopolysaccharide (LPS) treated group. Proteins were extracted, separated based on their isoelectric point and molecular weight, and visualized using silver staining. This gel map shows the protein expression profile of the LPS group compared to the control group, highlighting differentially expressed proteins. (B) 2‐DE gel map of hippocampal proteins from the control (CON) group. This map serves as a reference to identify changes in protein expression induced by LPS treatment. Proteins were extracted and separated under the same conditions as in the LPS group to ensure comparability. (C) Analysis of the significantly altered proteins identified in the 2‐DE gels. Gene Ontology (GO) analysis was performed to categorize these proteins into biological processes (BPs), cellular components (CCs), and molecular functions (MFs). The bar graph displays the top‐ranked categories with their respective *p* values, indicating the statistical significance of the enrichment. This analysis provides insights into the functional implications of the differential protein expression. (D) Pathway enrichment analysis of the significantly altered proteins. Proteins were mapped to KEGG pathways to identify significantly enriched pathways. Pathways with a false discovery rate (FDR) less than 0.05 were considered significantly altered, suggesting that they play a crucial role in the biological response to LPS treatment. The bubble diagram shows these pathways along with their respective *p* values and FDR values, highlighting the most affected biological processes.

Silver‐stained gels for the LPS and CON groups are shown in Figure 3A,B, respectively. A total of 106 differentially expressed protein points were identified on the basis of the criteria of 1.5‐fold. Then these protein spots were analyzed by mass spectrometry, resulting in 81 differential proteins. Details of these differential proteins are presented in Table 2. The top five most significantly upregulated proteins included Hadhb, Cdk5, Lyn, Cdk1, and Prdx6, with fold changes ranging from 3.41 to 5.06. Conversely, the top five most significantly downregulated proteins were Snap25, Psma5, Calb1, Rab3d, and Rab3a, with fold changes ranging from 0.16 to 0.21.

**TABLE 2:** Differentially expressed protein spots of lipopolysaccharide (LPS) and control (CON) group mice.

Spot ID	UniProt ID	Protein names	Gene names	Fold change (LPS/Dep)	Biological context
6	P12658	Calbindin	Calb1	0.21	Buffers cytosolic calcium. May stimulate a membrane Ca2+‐ATPase and a 3′,5′‐cyclic nucleotide phosphodiesterase
6	P35276	Ras‐related protein Rab‐3D	Rab3d	0.21	Protein transport. Probably involved in vesicular traffic (By similarity). May be involved in the insulin‐induced exocytosis of glut4‐containing vesicles in adipocytes
6	P63011	Ras‐related protein Rab‐3A	Rab3a	0.21	Small GTP‐binding protein that plays a central role in regulated exocytosis and secretion. Controls the recruitment, tethering and docking of secretory vesicles to the plasma membrane
15	Q61171	Peroxiredoxin‐2	Prdx2	0.27	Thiol‐specific peroxidase that catalyzes the reduction of hydrogen peroxide and organic hydroperoxides to water and alcohols, respectively. Plays a role in cell protection against oxidative stress by detoxifying peroxides and as sensor of hydrogen peroxide‐mediated signaling events. Might participate in the signaling cascades of growth factors and tumor necrosis factor‐alpha by regulating the intracellular concentrations of H_2_O_2_
17	Q99PT1	Rho GDP‐dissociation inhibitor 1	Arhgdia	0.28	Controls Rho proteins homeostasis. Regulates the GDP/GTP exchange reaction of the Rho proteins by inhibiting the dissociation of GDP from them, and the subsequent binding of GTP to them. Retains Rho proteins such as CDC42, RAC1, and RHOA in an inactive cytosolic pool, regulating their stability and protecting them from degradation. Actively involved in the recycling and distribution of activated Rho GTPases in the cell, mediates extraction from membranes of both inactive and activated molecules due its exceptionally high affinity for prenylated forms. Through the modulation of Rho proteins, may play a role in cell motility regulation. In glioma cells, inhibits cell migration and invasion by mediating the signals of SEMA5A and PLXNB3 that lead to inactivation of RAC1
39	P60879	Synaptosomal‐associated protein 25	Snap25	0.16	t‐SNARE involved in the molecular regulation of neurotransmitter release (PubMed:8103915, PubMed:8243676) May play an important role in the synaptic function of specific neuronal systems. Associates with proteins involved in vesicle docking and membrane fusion. Regulates plasma membrane recycling through its interaction with CENPF (PubMed:16672379) Modulates the gating characteristics of the delayed rectifier voltage‐dependent potassium channel KCNB1 in pancreatic beta cells (By similarity)
39	Q9Z2U1	Proteasome subunit alpha type‐5	Psma5	0.16	Component of the 20S core proteasome complex involved in the proteolytic degradation of most intracellular proteins. This complex plays numerous essential roles within the cell by associating with different regulatory particles. Associated with two 19S regulatory particles, forms the 26S proteasome and thus participates in the ATP‐dependent degradation of ubiquitinated proteins. The 26S proteasome plays a key role in the maintenance of protein homeostasis by removing misfolded or damaged proteins that could impair cellular functions, and by removing proteins whose functions are no longer required. Associated with the PA200 or PA28, the 20S proteasome mediates ubiquitin‐independent protein degradation. This type of proteolysis is required in several pathways including spermatogenesis (20S‐PA200 complex) or generation of a subset of MHC class I‐presented antigenic peptides (20S‐PA28 complex)
101	Q8R5L1	Complement component 1 Q subcomponent‐binding protein, mitochondrial	C1qbp	0.59	NA
109	P48036	Annexin A5	Anxa5	0.39	This protein is an anticoagulant protein that acts as an indirect inhibitor of the thromboplastin‐specific complex, which is involved in the blood coagulation cascade
110	Q8BGB7	Enolase‐phosphatase E1	Enoph1	0.3	Bifunctional enzyme that catalyzes the enolization of 2,3‐diketo‐5‐methylthiopentyl‐1‐phosphate (DK‐MTP‐1‐P) into the intermediate 2‐hydroxy‐3‐keto‐5‐methylthiopentenyl‐1‐phosphate (HK‐MTPenyl‐1‐P), which is then dephosphorylated to form the acireductone 1,2‐dihydroxy‐3‐keto‐5‐methylthiopentene (DHK‐MTPene)
121	Q61166	Microtubule‐associated protein RP/EB family member 1	Mapre1	0.4	Plus‐end tracking protein (+TIP) that binds to the plus‐end of microtubules and regulates the dynamics of the microtubule cytoskeleton. Recruits other +TIP proteins to microtubules by binding to a conserved Ser‐X‐Leu‐Pro (SXLP) motif in their polypeptide chains
133	O70251	Elongation factor 1‐beta	Eef1b	0.33	EF‐1‐beta and EF‐1‐delta stimulate the exchange of GDP bound to EF‐1‐alpha to GTP
133	P21107	Tropomyosin alpha‐3 chain	Tpm3	0.33	Binds to actin filaments in muscle and non‐muscle cells. Plays a central role, in association with the troponin complex, in the calcium dependent regulation of vertebrate striated muscle contraction. Smooth muscle contraction is regulated by interaction with caldesmon. In non‐muscle cells is implicated in stabilizing cytoskeleton actin filaments
891	Q8R0Y6	Cytosolic 10‐formyltetrahydrofolate dehydrogenase	Aldh1l1	1.65	Cytosolic 10‐formyltetrahydrofolate dehydrogenase that catalyzes the NADP+‐dependent conversion of 10‐formyltetrahydrofolate to tetrahydrofolate and carbon dioxide
1021	Q9DCX2	ATP synthase subunit d, mitochondrial	Atp5pd	0.59	Mitochondrial membrane ATP synthase (F1F0 ATP synthase or Complex V) produces ATP from ADP in the presence of a proton gradient across the membrane which is generated by electron transport complexes of the respiratory chain
1021	Q9R0Y5	Adenylate kinase isoenzyme 1	Ak1	0.59	Catalyzes the reversible transfer of the terminal phosphate group between ATP and AMP. Also displays broad nucleoside diphosphate kinase activity
1034	P70296	Phosphatidylethanolamine‐binding protein 1	Pebp1	0.63	Binds ATP, opioids and phosphatidylethanolamine. Has lower affinity for phosphatidylinositol and phosphatidylcholine
1224	P68369	Tubulin alpha‐1A chain	Tuba1a	0.35	Tubulin is the major constituent of microtubules, a cylinder consisting of laterally associated linear protofilaments composed of alpha‐ and beta‐tubulin heterodimers
2171	O35295	Transcriptional activator protein Pur‐beta	Purb	0.64	Transcriptional regulator which can act as an activator or a repressor
2171	P18872	Guanine nucleotide‐binding protein G(o) subunit alpha	Gnao1	0.64	Guanine nucleotide‐binding proteins (G proteins) function as transducers downstream of G protein‐coupled receptors (GPCRs) in numerous signaling cascades
2301	Q60854	Serpin B6	Serpinb6	0.47	Inhibitor of cathepsin G, kallikrein‐8, and thrombin. May play an important role in the inner ear in the protection against leakage of lysosomal content during stress. May be involved in the regulation of serine proteinases present in the brain or extravasated from the blood
2301	Q8BFZ9	Erlin‐2	Erlin2	0.47	Component of the ERLIN1/ERLIN2 complex which mediates the endoplasmic reticulum‐associated degradation (ERAD) of inositol 1,4,5‐trisphosphate receptors (IP3Rs) such as ITPR1
2503	Q61696	Heat shock 70 kDa protein 1A	Hspa1a	0.44	Molecular chaperone implicated in a wide variety of cellular processes, including protection of the proteome from stress, folding and transport of newly synthesized polypeptides, activation of proteolysis of misfolded proteins and the formation and dissociation of protein complexes
2503	Q9WUM3	Coronin‐1B	Coro1b	0.44	Regulates leading edge dynamics and cell motility in fibroblasts. May be involved in cytokinesis and signal transduction
2677	Q9DBS2	Tumor protein p63‐regulated gene 1‐like protein	Tprg1l	0.55	Presynaptic protein involved in the synaptic transmission tuning. Regulates synaptic release probability by decreasing the calcium sensitivity of release
2925	Q9CQF3	Cleavage and polyadenylation specificity factor subunit 5	Nudt21	0.5	Component of the cleavage factor Im (CFIm) complex that functions as an activator of the pre‐mRNA 3′‐end cleavage and polyadenylation processing required for the maturation of pre‐mRNA into functional mRNAs
3009	O08709	Peroxiredoxin‐6	Prdx6	3.41	Thiol‐specific peroxidase that catalyzes the reduction of hydrogen peroxide and organic hydroperoxides to water and alcohols, respectively
3304	Q04447	Creatine kinase B‐type	Ckb	0.32	Reversibly catalyzes the transfer of phosphate between ATP and various phosphogens
4116	Q8BLJ3	PI‐PLC X domain‐containing protein 3	Plcxd3	2.57	NA
4420	O89053	Coronin‐1A	Coro1a	1.76	May be a crucial component of the cytoskeleton of highly motile cells, functioning both in the invagination of large pieces of plasma membrane, as well as in forming protrusions of the plasma membrane involved in cell locomotion
4505	O08553	Dihydropyrimidinase‐related protein 2	Dpysl2	1.57	Plays a role in neuronal development and polarity, as well as in axon growth and guidance, neuronal growth cone collapse and cell migration. Necessary for signaling by class 3 semaphorins and subsequent remodeling of the cytoskeleton. May play a role in endocytosis
4521	Q9QXY6	EH domain‐containing protein 3	Ehd3	0.57	ATP‐ and membrane‐binding protein that controls membrane reorganization/tubulation upon ATP hydrolysis. In vitro causes tubulation of endocytic membranes
4713	Q91W50	Cold shock domain‐containing protein E1	Csde1	0.65	RNA‐binding protein involved in translationally coupled mRNA turnover. Implicated with other RNA‐binding proteins in the cytoplasmic deadenylation/translational and decay interplay of the FOS mRNA mediated by the major coding‐region determinant of instability (mCRD) domain. Required for efficient formation of stress granules
5103	Q8K354	Carbonyl reductase [NADPH] 3	Cbr3	1.64	Catalyzes the NADPH‐dependent reduction of carbonyl compounds to their corresponding alcohols. Has low NADPH‐dependent oxidoreductase activity. Acts on several orthoquinones, as well as on non‐quinone compounds, such as isatin or on the anticancer drug oracin. Best substrates for CBR3 is 1,2‐naphthoquinone, hence could play a role in protection against cytotoxicity of exogenous quinones. Exerts activity toward ortho‐quinones but not paraquinones. No endogenous substrate for CBR3 except isatin has been identified
5209	Q91YR1	Twinfilin‐1	Twf1	1.56	Actin‐binding protein involved in motile and morphological processes. Inhibits actin polymerization, likely by sequestering G‐actin. By capping the barbed ends of filaments, it also regulates motility. Seems to play an important role in clathrin‐mediated endocytosis and distribution of endocytic organelles
5209	Q9Z0P5	Twinfilin‐2	Twf2	1.56	Actin‐binding protein involved in motile and morphological processes. Inhibits actin polymerization, likely by sequestering G‐actin. By capping the barbed ends of filaments, it also regulates motility. Seems to play an important role in clathrin‐mediated endocytosis and distribution of endocytic organelles. May play a role in regulating the mature length of the middle and short rows of stereocilia
5212	P05063	Fructose‐bisphosphate aldolase C	Aldoc	1.67	NA
5212	P60335	Poly(rC)‐binding protein 1	Pcbp1	1.67	Single‐stranded nucleic acid binding protein that binds preferentially to oligo dC
5215	Q93092	Transaldolase	Taldo1	2.66	Catalyzes the rate‐limiting step of the non‐oxidative phase in the pentose phosphate pathway. Catalyzes the reversible conversion of sedheptulose‐7‐phosphate and d‐glyceraldehyde 3‐phosphate into erythrose‐4‐phosphate and beta‐d‐fructose 6‐phosphate
5218	D3Z3N4	Heterogeneous nuclear ribonucleoprotein H3	Hnrnph3	2.01	NA
5218	Q8K0S0	Phytanoyl‐CoA hydroxylase‐interacting protein	Phyhip	2.01	Its interaction with PHYH suggests a role in the development of the central system
5307	Q9JHI5	Isovaleryl‐CoA dehydrogenase, mitochondrial	Ivd	1.94	Catalyzes the conversion of isovaleryl‐CoA/3‐methylbutanoyl‐CoA to 3‐methylbut‐2‐enoyl‐CoA as an intermediate step in the leucine (Leu) catabolic pathway. To a lesser extent, is also able to catalyze the oxidation of other saturated short‐chain acyl‐CoA thioesters as pentanoyl‐CoA, hexenoyl‐CoA, and butenoyl‐CoA
5307	Q9Z2Q6	Septin‐5	Septin5	1.94	Filament‐forming cytoskeletal GTPase
5313	Q99L15	Acot1 protein	Acot1	0.37	NA
5313	Q9QYR9	Acyl‐coenzyme A thioesterase 2, mitochondrial	Acot2	0.37	Catalyzes the hydrolysis of acyl‐CoAs into free fatty acids and coenzyme A (CoASH), regulating their respective intracellular levels
5317	Q61553	Fascin	Fscn1	2.06	Actin‐binding protein that contains two major actin binding sites
5601	P26043	Radixin	Rdx	0.36	Probably plays a crucial role in the binding of the barbed end of actin filaments to the plasma membrane
5617	Q3THK7	GMP synthase [glutamine‐hydrolyzing]	Gmps	1.96	Catalyzes the conversion of xanthine monophosphate (XMP) to GMP in the presence of glutamine and ATP through an adenyl‐XMP intermediate
5617	Q64332	Synapsin‐2	Syn2	1.96	Neuronal phosphoprotein that coats synaptic vesicles, binds to the cytoskeleton, and is believed to function in the regulation of neurotransmitter release. May play a role in noradrenaline secretion by sympathetic neurons
6002	Q60932	Voltage‐dependent anion‐selective channel protein 1	Vdac1	1.85	Non‐selective voltage‐gated ion channel that mediates the transport of anions and cations through the mitochondrion outer membrane and plasma membrane
6014	Q80W21	Glutathione *S*‐transferase Mu 7	Gstm7	0.32	Conjugation of reduced glutathione to a wide number of exogenous and endogenous hydrophobic electrophiles
6110	O88601	Syntenin	Sdcbp	1.74	NA
6202	Q3ULJ0	Glycerol‐3‐phosphate dehydrogenase 1‐like protein	Gpd1l	2.2	Plays a role in regulating cardiac sodium current; decreased enzymatic activity with resulting increased levels of glycerol 3‐phosphate activating the DPD1L‐dependent SCN5A phosphorylation pathway, may ultimately lead to decreased sodium current; cardiac sodium current may also be reduced due to alterations of NAD(H) balance induced by DPD1L
6207	P05201	Aspartate aminotransferase, cytoplasmic	Got1	1.77	Biosynthesis of l‐glutamate from l‐aspartate or l‐cysteine. Important regulator of levels of glutamate, the major excitatory neurotransmitter of the vertebrate central nervous system
6308	A0A2R8W6V9	Neuronal‐specific septin‐3	Septin3	2.03	NA
6318	Q91Z53	Glyoxylate reductase/Hydroxypyruvate reductase	Grhpr	1.55	Enzyme with hydroxy‐pyruvate reductase, glyoxylate reductase and d‐glycerate dehydrogenase enzymatic activities. Reduces hydroxypyruvate to d‐glycerate, glyoxylate to glycolate oxidizes d‐glycerate to hydroxypyruvate
6418	Q80XI4	Phosphatidylinositol 5‐phosphate 4‐kinase type‐2 beta	Pip4k2b	1.66	Participates in the biosynthesis of phosphatidylinositol 4,5‐bisphosphate
6418	Q8BWF0	Succinate‐semialdehyde dehydrogenase, mitochondrial	Aldh5a1	1.66	Catalyzes one step in the degradation of the inhibitory neurotransmitter gamma‐aminobutyric acid (GABA)
7109	P68040	Receptor of activated protein C kinase 1	Rack1	1.95	Scaffolding protein involved in the recruitment, assembly and/or regulation of a variety of signaling molecules
7109	Q4FZG5	Arp2/3 complex 34 kDa subunit	Arpc2	1.95	Actin‐binding component of the Arp2/3 complex, a multiprotein complex that mediates actin polymerization upon stimulation by nucleation‐promoting factor (NPF)
7109	Q91V64	Isochorismatase domain‐containing protein 1	Isoc1	1.95	NA
7109	Q91VH6	Protein MEMO1	Memo1	1.95	May control cell migration by relaying extracellular chemotactic signals to the microtubule cytoskeleton. Mediator of ERBB2 signaling
7115	P38060	Hydroxymethylglutaryl‐CoA lyase, mitochondrial	Hmgcl	2.26	Mitochondrial 3‐hydroxy‐3‐methylglutaryl‐CoA lyase that catalyzes a cation‐dependent cleavage of (*S*)‐3‐hydroxy‐3‐methylglutaryl‐CoA into acetyl‐CoA and acetoacetate, a key step in ketogenesis
7115	Q91W61	F‐box/LRR‐repeat protein 15	Fbxl15	2.26	Substrate recognition component of a SCF (SKP1‐CUL1‐F‐box protein) E3 ubiquitin‐protein ligase complex which mediates the ubiquitination and subsequent proteasomal degradation of SMURF1, thereby acting as a positive regulator of the BMP signaling pathway
7217	Q8C0M9	Isoaspartyl peptidase/l‐Asparaginase	Asrgl1	3.28	Has both l‐asparaginase and beta‐aspartyl peptidase activity
7311	P97807	Fumarate hydratase, mitochondrial	Fh	1.67	Catalyzes the reversible stereospecific interconversion of fumarate to l‐malate
7311	Q9DCD0	6‐Phosphogluconate dehydrogenase, decarboxylating	Pgd	1.67	Catalyzes the oxidative decarboxylation of 6‐phosphogluconate to ribulose 5‐phosphate and CO_2_, with concomitant reduction of NADP to NADPH
7311	Q9QUP5	Hyaluronan and proteoglycan link protein 1	Hapln1	1.67	Stabilizes the aggregates of proteoglycan monomers with hyaluronic acid in the extracellular cartilage matrix
7408	P61922	4‐Aminobutyrate aminotransferase, mitochondrial	Abat	1.69	Catalyzes the conversion of gamma‐aminobutyrate and l‐beta‐aminoisobutyrate to succinate semialdehyde and methylmalonate semialdehyde, respectively. Can also convert delta‐aminovalerate and beta‐alanine
7532	P52480	Pyruvate kinase PKM	Pkm	2.84	Catalyzes the final rate‐limiting step of glycolysis by mediating the transfer of a phosphoryl group from phosphoenolpyruvate (PEP) to ADP, generating ATP
8001	P16858	Glyceraldehyde‐3‐phosphate dehydrogenase	Gapdh	1.6	Has both glyceraldehyde‐3‐phosphate dehydrogenase and nitrosylase activities, thereby playing a role in glycolysis and nuclear functions, respectively
8001	Q8R0F8	Acylpyruvase FAHD1, mitochondrial	Fahd1	1.6	Tautomerase that converts enol‐oxaloacetate, a strong inhibitor of succinate dehydrogenase, to the physiological keto form of oxaloacetate
8010	P17751	Triosephosphate isomerase	Tpi1	2.06	Triosephosphate isomerase is an extremely efficient metabolic enzyme that catalyzes the interconversion between dihydroxyacetone phosphate (DHAP) and d‐glyceraldehyde‐3‐phosphate (G3P) in glycolysis and gluconeogenesis
8010	Q9D172	Glutamine amidotransferase‐like class 1 domain‐containing protein 3, mitochondrial	Gatd3	2.06	NA
8101	P11440	Cyclin‐dependent kinase 1	Cdk1	4.05	Plays a key role in the control of the eukaryotic cell cycle by modulating the centrosome cycle as well as mitotic onset; promotes G2‐M transition via association with multiple interphase cyclins
8101	P25911	Tyrosine‐protein kinase Lyn	Lyn	4.05	Non‐receptor tyrosine‐protein kinase that transmits signals from cell surface receptors and plays an important role in the regulation of innate and adaptive immune responses, hematopoiesis, responses to growth factors and cytokines, integrin signaling, but also responses to DNA damage and genotoxic agents
8101	P49615	Cyclin‐dependent‐like kinase 5	Cdk5	4.05	Proline‐directed serine/threonine‐protein kinase essential for neuronal cell cycle arrest and differentiation and may be involved in apoptotic cell death in neuronal diseases by triggering abortive cell cycle re‐entry
8116	Q3TDK6	Protein rogdi homolog	Rogdi	1.55	NA
8133	Q9Z2U0	Proteasome subunit alpha type‐7	Psma7	1.81	Component of the 20S core proteasome complex involved in the proteolytic degradation of most intracellular proteins
9135	O55125	Protein NipSnap homolog 1	Nipsnap1	1.63	Protein involved in mitophagy by facilitating recruitment of the autophagy machinery required for clearance of damaged mitochondria
9306	Q99JY0	Trifunctional enzyme subunit beta, mitochondrial	Hadhb	5.06	Mitochondrial trifunctional enzyme catalyzes the last three of the four reactions of the mitochondrial beta‐oxidation pathway

*Note*: Proteins with fold changes >1 and <1 are significantly increased and decreased, respectively.

We further employed GO analysis and KEGG pathway analysis to reveal the biological functions involved in the differential proteins. GO analysis showed that a total of 267 BP terms, 59 CC terms, and 62 MF terms were significantly enriched (all FDR < 0.05). The top‐ranked GO terms are shown in Figure 3C. Among these terms, purine ribonucleotide metabolic process and cellular amino acid catabolic process are top‐ranked BP terms.

The results of pathway analysis identified a total of 20 nominally significantly enriched pathways. After FDR correction, 8 pathways remained significantly enriched (FDR < 0.05, Figure 3D). Among these enriched pathways, “valine, leucine and isoleucine degradation,” “alanine, aspartate and glutamate metabolism,” and “biosynthesis of amino acids” were amino acid‐related metabolic pathways, suggesting that LPS can significantly alter amino acid metabolism in the hippocampus.

### 3.4 Effects of LPS on the Hippocampal Gene Expressions of Mice

Using PCR microarray, we analyzed the expression of 84 genes related to neuropsychiatric diseases. Four significantly altered mRNAs were obtained (Table 3). Among them, the expression levels of Aldoc (FC = 1.26, *p* = 0.042), Lyn (FC = 2.13, *p* < 0.001), and Prodh (FC = 1.44, *p* = 0.017) were significantly increased, whereas Asrgl1 (FC = 0.76, *p* = 0.050) was significantly reduced. Interestingly, three of these genes (Aldoc, Lyn, and Asrgl1) were also differentially altered at the protein level.

**TABLE 3:** Differential expressed genes of lipopolysaccharide (LPS) and control (CON) group mice.

Gene symbol	Fold change (LPS/Dep)	*p* value
Asrgl1	0.76	0.050
Aldoc	1.26	0.042
Lyn	2.13	<0.001
Prodh	1.44	0.017

### 3.5 Joint Pathway Analysis Based On Differential Metabolites, Proteins, and Genes

**FIGURE 4:**
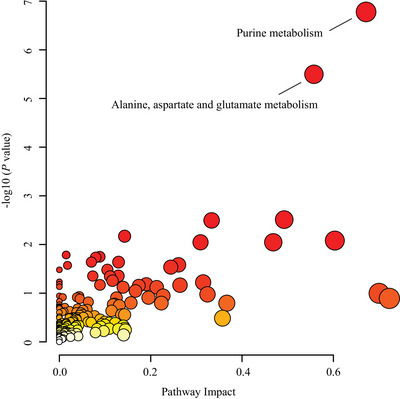
Joint pathway analysis based on differential metabolites, proteins, and genes. The names of significantly enriched pathways (FDR < 0.05) are shown. The enrichment of pathways such as purine metabolism and alanine, aspartate, and glutamate metabolism suggests alterations in key metabolic processes that could influence neuronal function and behavior.

We further explored the biological functions of differential molecules by integration analysis. On the basis of differential metabolites, proteins, and genes, a total of 21 nominally differential KEGG pathways were obtained. After FDR correction, only two pathways were still significantly enriched, including purine metabolism (FDR < 0.001) and alanine, aspartate, and glutamate metabolism (FDR = 0.001; Figure 4). Furthermore, through additional analysis with IPA, we confirmed that the purine metabolism pathway remained significantly enriched after FDR correction (FDR < 0.001). This result highlights the potential key role of purine metabolism in the context of our study and supports our understanding of the biological functions of differential molecules (FDR < 0.001; Figure S2). In addition to the well‐known pathways, our analysis identified several novel pathway interactions. For instance, the glycolysis or gluconeogenesis pathway was identified as a nominally significant pathway (*p* < 0.05) before FDR correction, suggesting a potential role in LPS‐induced depression‐like behaviors. This finding aligns with previous studies suggesting that alterations in glucose metabolism may contribute to the pathophysiology of depression (Ernst et al. 2017; Yao et al. 2023). Our results build upon these findings by demonstrating a potential role for this pathway in LPS‐induced depression‐like behaviors, thereby reinforcing its relevance as a therapeutic target. This pathway's significance in our study is further highlighted by the lack of FDR correction, which underscores the need for further investigation into its role in depression and the potential for developing novel interventions.

**FIGURE 5:**
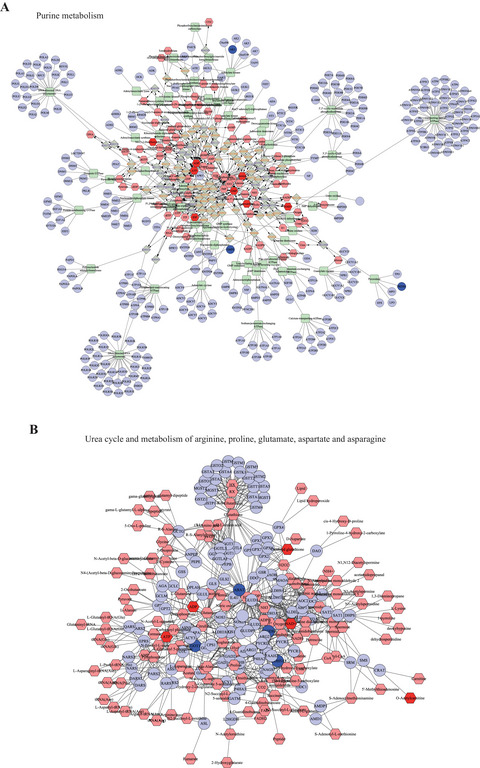
Compound‐reaction‐enzyme‐gene network plot based on the significantly enriched pathways of differential metabolites, proteins, and genes. (A) Compound‐reaction‐enzyme‐gene network based on the “purine metabolism” pathway. (B) Compound‐reaction‐enzyme‐gene network based on the “urea cycle and metabolism of arginine, proline, glutamate, aspartate, and asparagine” pathway. This network was generated using MetScape software, which integrates differential metabolites, proteins, and genes to visualize their interactions and regulatory relationships. In this network, squares represent enzymes, circles represent genes, and hexagons denote compounds. The edges between nodes indicate the reactions or regulatory interactions between them.

**FIGURE 6:**
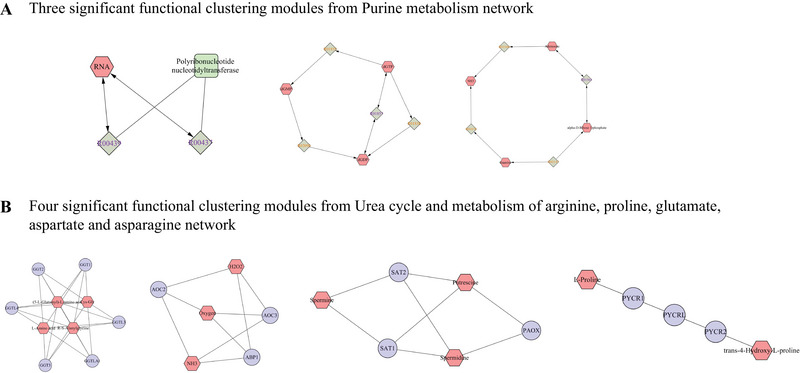
Significant functional modules from compound‐reaction‐enzyme‐gene networks. (A) Three significant functional clustering modules from Purine metabolism network. (B) Four significant functional clustering modules from Urea cycle and metabolism of arginine, proline, glutamate, aspartate, and asparagine network.

Subsequently, we used MetScape to construct compound‐reaction‐enzyme‐gene networks. “Purine metabolism” and “urea cycle and metabolism of arginine, proline, glutamate, aspartate, and asparagine” were set as target pathways. The constructed networks are shown in Figure 5A,B. Using the MCODE plugin in Cytoscape, we then identified three and four tightly connected functional modules from these networks, respectively, as illustrated in Figure 6A,B.

## 4 Discussion

Neuronal and immune systems interact, causing neuroinflammation (Kabata and Artis 2019). Inflammation is associated with depression, but its mechanism is unclear. We analyzed the hippocampus of LPS‐induced depressive‐like mice and identified 44 metabolites, 81 proteins, and 4 genes related to purine and glutamate metabolism disturbances. This is the first multi‐omics study exploring how acute LPS affects hippocampal molecules, leading to depression‐like behaviors. Our study revealed possible pathogenic mechanisms of acute inflammation‐induced depression.

Metabolomics has been widely used to explore the molecular mechanisms of depression (Pu, Liu, Zhang, et al., 2021; Pu et al. 2020). In the present study, we used the metabolomic approach to explore the relationship between hippocampal metabolomic alterations and depression‐like behaviors. Among the differential metabolites identified, oxidized glutathione stood out as a notable finding in both positive and negative ion modes. Oxidized glutathione is a derivative of glutathione, which is an important antioxidant in cellular redox balance. It is involved in the protection against oxidative stress and can modulate the cellular response to reactive oxygen species (Lv et al. 2019). Our findings indicate that alterations in purine metabolism are associated with depression‐like behaviors. Notably, the nominal significance of the glycolysis or gluconeogenesis pathway (*p* < 0.05) before FDR correction suggests potential alterations in glucose metabolism. This pathway, which is integral to energy metabolism, may play a pivotal role in the response to LPS‐induced inflammation (Ernst et al. 2017).

A study found that purine metabolism altered in LPS‐treated rats’ hippocampus (Geng et al. 2019), aligning with our results. Moreover, it has been confirmed that metabolic disturbances due to abnormal neuroinflammation mediated the onset of depression by the detection of hypothalamic metabolites in LPS model mice. The metabolite disorders mainly focus on purine metabolism, in which glutamine, as a key metabolite involved in both amino acid and purine metabolism, plays an important role in the dysfunction of purinergic system and imbalance of glutamatergic system in depressed patients (Wu et al. 2017). Furthermore, the differential metabolites identified in the current study are different from differential metabolites found in the hippocampus of classical stress models (Pu, Liu, Gui, et al., 2021), suggesting that metabolic alterations caused by different pathogenic factors may be inconsistent.

In this study, we obtained a series of differential metabolites involved in purine metabolism, including adenosine triphosphate (ATP), adenosine diphosphate (ADP), N6‐(1,2‐dicarboxyethyl)‐AMP, inosine diphosphate, guanosine monophosphate, hypoxanthine, inosine, and ADP‐ribose. These metabolites play important roles in the regulation of intracellular energy homeostasis (Huang et al. 2021). Notably, ATP, ADP, and nicotinamide adenine dinucleotide increased. This may be due to systemic inflammation causing hippocampal energy expenditure (Jones et al. 2010). Increased ATP levels could also be a brain cell response to systemic inflammatory injury, activating stress‐related signaling pathways (Fiebich et al. 2014).

It has been found that after depression treatment, the target of cure was focused on purine metabolism, and by targeting quantitative analysis of purine metabolites in the cortex, it was confirmed that excessive synthesis of the purine metabolite xanthine may mediate activation of the NLRP3 inflammatory vesicle pathway, leading to depression (Chen et al. 2022; Ji et al. 2022).

We studied LPS's impact on hippocampal molecular profiles using proteomics and PCR microarrays, finding 81 differential proteins and four genes. Three genes matched proteomic data, validating our findings. The upregulation of proteins such as Hadhb, Cdk5, Lyn, Cdk1, and Prdx6, alongside the downregulation of Snap25, Psma5, Calb1, Rab3d, and Rab3a, provides insights into the molecular mechanisms underlying depression‐like behaviors induced by LPS. The substantial upregulation of Hadhb, a crucial component of the tricarboxylic acid cycle (Dagher et al. 2021), indicates an enhanced energy metabolism, potentially serving as a compensatory mechanism to meet the heightened energy demands of neurons during inflammation, which could reflect the brain's efforts to adapt to metabolic stress imposed by LPS. Additionally, the increased expression of Cdk5 and Cdk1, cyclin‐dependent kinases (Malumbres 2014), suggests their role in cell cycle regulation, crucial for modulating cell proliferation and survival under inflammatory stress, potentially aiding neuronal adaptation to stress and inflammation. Furthermore, the upregulation of Lyn, a non‐receptor tyrosine kinase involved in immune cell signaling (Ben‐Khemis et al. 2023), may contribute to the activation of inflammatory pathways in the hippocampus, potentially initiating or exacerbating depressive‐like behaviors through inflammatory signaling cascades. Lastly, the increased expression of Prdx6, an antioxidant enzyme, indicates an adaptive response to oxidative stress (Jia et al. 2023), a common feature of neuroinflammatory conditions (Mingoti et al. 2022), potentially serving as a protective mechanism against oxidative damage implicated in the pathophysiology of depression (Choi et al. 2024). Conversely, the downregulation of Snap25, involved in synaptic vesicle formation and neurotransmitter release (Herreros et al. 1997), and Psma5 and Calb1, which are part of the proteasome and play roles in protein degradation and synaptic plasticity (Livinskaya et al. 2012; Soontornniyomkij et al. 2012), respectively, may impair synaptic function and contribute to depressive‐like behaviors. The downregulation of Rab3d and Rab3a, proteins involved in vesicular transport and neurotransmitter release (Raffaniello 2021), could affect neurotransmission efficiency and synaptic plasticity, potentially leading to mood disorders.

GO and KEGG analyses showed significant changes in alanine, aspartate, and glutamate metabolism, indicating abnormal amino acid metabolism in the hippocampus due to LPS. Integration analysis also suggested alterations in this pathway, involving four upregulated proteins: Got1, Aldh5a1, Asrgl1, and Abat. Got1 regulates glutamate metabolism and has neuroprotective effects (Dopico‐López et al. 2021; Guo et al. 2016). Aldh5a1 and Abat are key in gamma‐aminobutyric acid (GABA) catabolism and mitochondrial nucleoside metabolism (Besse et al. 2015; Kim et al. 2011). Other studies showed that LPS increases glutamate and GABA in the hippocampus (Geng et al. 2019; Guo et al. 2016). Evidence suggests that systemic inflammation disturbs glutamate metabolism in the hippocampus, though the mechanism is unclear. One reason could be increased neurotoxic quinolinic acid due to inflammation, raising glutamate levels (Müller and Schwarz 2007; Zhang et al. 2020). Inflammation also affects glutamate balance in nerves, astrocytes, oligodendrocytes, and microglia (Haroon et al. 2014; Liu et al. 2020). Inflammation and altered glutamate metabolism are linked to depression pathogenesis, with increased inflammation in depressed patients leading to higher glutamate in the basal ganglia and glial dysfunction (Haroon et al. 2016; Haroon and Miller 2017). Increased basal ganglia glutamate in depression is associated with anhedonia and psychomotor retardation, whereas increased inflammatory markers like C‐reactive protein correlate with higher glutamate, indicating that inflammation affects glutamate metabolism in depression (Haroon and Miller 2017).

In the current study, metabolomics analysis found 10 differential lipids, including phosphatidylcholine, phosphatidylglycerol, phosphatidylinositol, and lysophosphatidylethanolamine. A prior study reported LPS‐induced lipid metabolism abnormalities in the hippocampus alleviated by antidepressants (Geng, Hao, et al. 2020). Pathway analysis indicated amino acid metabolism alterations involving Acot1, Acot2, and Hadhb, all involved in mitochondrial beta‐oxidation. Acot1/2 hydrolyzes fatty acyl‐CoA esters to reduce beta‐oxidation overload (Bekeova et al. 2019; Liu et al. 2021), whereas LPS activates Hadhb, enhancing mitochondrial β‐oxidation and regulating oxylipins (Misheva et al. 2022). Additionally, acetylcarnitine and butyryl‐l‐carnitine concentrations increased, known for their antidepressant and neuroprotective effects via β‐oxidation (Ferreira and McKenna 2017; Nasca et al. 2018). These findings suggest systemic inflammation disrupts lipid metabolism and increases β‐oxidation in the CNS, with mechanisms needing further exploration.

Chronic inflammation models, in comparison to acute inflammation models, exhibit more complex and sustained modifications in purine and glutamate metabolism (Linden et al. 2019; Zizmare et al. 2022). These disturbances can lead to heightened uric acid concentrations, activating the NLRP3 inflammasome pathway and triggering inflammatory responses (Wu et al. 2024). Chronic inflammation also disrupts the glutamatergic system's balance, potentially causing extended glutamate accumulation and neurotoxic effects, which can affect neural functionality (Martínez‐Rizo et al. 2024). Additionally, chronic inflammation models show alterations in proteins and metabolites linked to long‐term immune activation and tissue remodeling, including changes in lipid metabolism, oxidative stress, and neurodegenerative pathways (Aiello et al. 2024; Zou et al. 2025). Although both acute and chronic models display disturbances in energy metabolism and purine metabolism, chronic models exhibit more extensive and complex changes, particularly in lipid metabolism and neurodegenerative pathways (characterized by prolonged inflammatory pathway activation) (Vanherle et al. 2025; Zhang et al. 2025). Our study suggests that targeting purine and glutamate metabolism pathways, such as ATP/ADP, xanthine, glutamate transporters, and NMDA receptors, may offer therapeutic potential for treating depression by reducing inflammation, oxidative stress, and excitotoxicity, with drugs like P2X7 receptor antagonists, allopurinol, riluzole, and ketamine showing promise in this regard (Bai et al. 2024; Cavaleri et al. 2024; Chrobak and Siwek 2024; Das et al. 2024; Fernandes et al. 2016). Furthermore, given the emerging literature on mechanisms underlying the antidepressant effects of trendy therapeutic options such as ketamine and esketamine (Cavaleri et al. 2024), and the potential of metabolomics in identifying novel therapeutic targets (Chaki and Watanabe 2023; Witkin et al. 2022), our findings could inform the development of next‐generation antidepressants, such as mGlu2/3 receptor antagonists.

The differential metabolites and proteins identified in our study, specifically those implicated in purine and glutamate metabolism, hold promise as potential biomarkers for diagnosing inflammation‐associated depression. These biomarkers could facilitate early detection and patient stratification, paving the way for more tailored treatment approaches. The involvement of purine and glutamate metabolism in LPS‐induced depression hints at these pathways as viable therapeutic targets. Modulating the activity of enzymes within these pathways or employing dietary interventions to rectify metabolic imbalances may alleviate depressive symptoms. This is consonant with emerging data on the antidepressant effects of metabolite‐directed therapies, such as the use of NMDAR antagonists like ketamine and esketamine, which also exert influence on glutamate signaling (Dogra and Conn 2021; Musazzi et al. 2013). Our insights into the role of neuroinflammation in depression could illuminate the path for the development of next‐generation antidepressants.

## 5 Limitation

The present study has several inherent limitations worth noting. First, our investigation was confined solely to the molecular alterations occurring in the hippocampus following acute inflammation. Consequently, it remains uncertain whether these molecular changes align with those observed under chronic inflammatory conditions. To elucidate the similarities and differences in molecular profiles between acute and chronic inflammation, further studies involving chronic LPS interventions are imperative.

Second, depression is recognized as a multifaceted disorder, yet our study solely examined the impact of inflammation on the hippocampal molecular profile. Despite employing rigorous statistical methodologies to pinpoint differential molecules and pathways, our study is not devoid of limitations. Notably, some analyses lacked multiple testing corrections, and our reliance on the comprehensiveness of databases is a concern. Although this study offers a comprehensive overview of molecular changes associated with LPS‐induced depressive‐like behavior through a multi‐omics approach, it is crucial to acknowledge the inherent constraints of the cross‐sectional study design.

Cross‐sectional studies inherently possess several limitations. First, they evaluate variables at a single point in time, rendering it impossible to discern the temporal sequence of events and challenging the establishment of causality. Second, such designs are prone to reverse causality, where the outcome variable may precede changes in the predictor variables. Additionally, cross‐sectional studies are unable to assess changes or trends over time, thereby limiting our understanding of the stability, variability, and long‐term patterns of variables.

To address these issues in future research, it is advisable to adopt more stringent statistical criteria and increase sample sizes, which would bolster the reliability of the identified pathways and molecular players.

## 6 Conclusion

Our study adopted a multi‐omics strategy to explore the molecular alterations of LPS intervention on depressive behavior and molecular characteristics of hippocampus in mice. We found that systemic inflammation can cause multidimensional molecular changes in the hippocampus of mice, and further biological function analysis suggested that disturbances of purine metabolism and glutamate metabolism are the main molecular features in the hippocampus of LPS‐induced depressive‐like mice. The findings of this study provide new insights into the molecular mechanisms of inflammation‐related depression.

## Author Contributions

**Wen‐Wen Li**: conceptualization, methodology, validation, software, data curation, investigation, funding acquisition. **Rui Xiao**: conceptualization, methodology, software, data curation, formal analysis, investigation, writing – review and editing. **Xue‐Yi Chen**: validation, formal analysis, software, resources, investigation, writing – review and editing. **Jun‐Cai Pu**: writing – original draft, conceptualization, methodology, validation, formal analysis, resources, writing – review and editing. **Jian‐Jun Chen**: conceptualization, investigation, methodology, validation. **Hai‐Yang Wang**: conceptualization, investigation, validation. **Lan‐Xiang Liu**: investigation, methodology, validation. **Dan Li**: investigation, visualization, project administration. **Yang‐Dong Zhang**: investigation, writing – review and editing, validation. **Wen‐Xia Li**: methodology, validation. **Peng Xie**: funding acquisition, supervision.

## Conflicts of Interest

The authors declare no conflicts of interest.

### Peer Review

The peer review history for this article is available at https://publons.com/publon/10.1002/brb3.70549.

## Supporting information



Supporting Information

Supporting Information

## Data Availability

The data that support the findings of this study are available on request from the corresponding author. The data are not publicly available due to privacy or ethical restrictions.
