# Supplementary material for: Multi‐Omics Analysis Reveals Disturbances of Purine Metabolism and Glutamate Metabolism in the Hippocampus of Lipopolysaccharide‐Induced Mouse Model of Depression
Source: Brain Behav. 2025 May 11;15(5):e70549. doi: 10.1002/brb3.70549 (PMC12066810; doi:10.1002/brb3.70549)
Supplement: Supplementary file 1 — Supporting Information [file BRB3-15-e70549-s002.pdf]

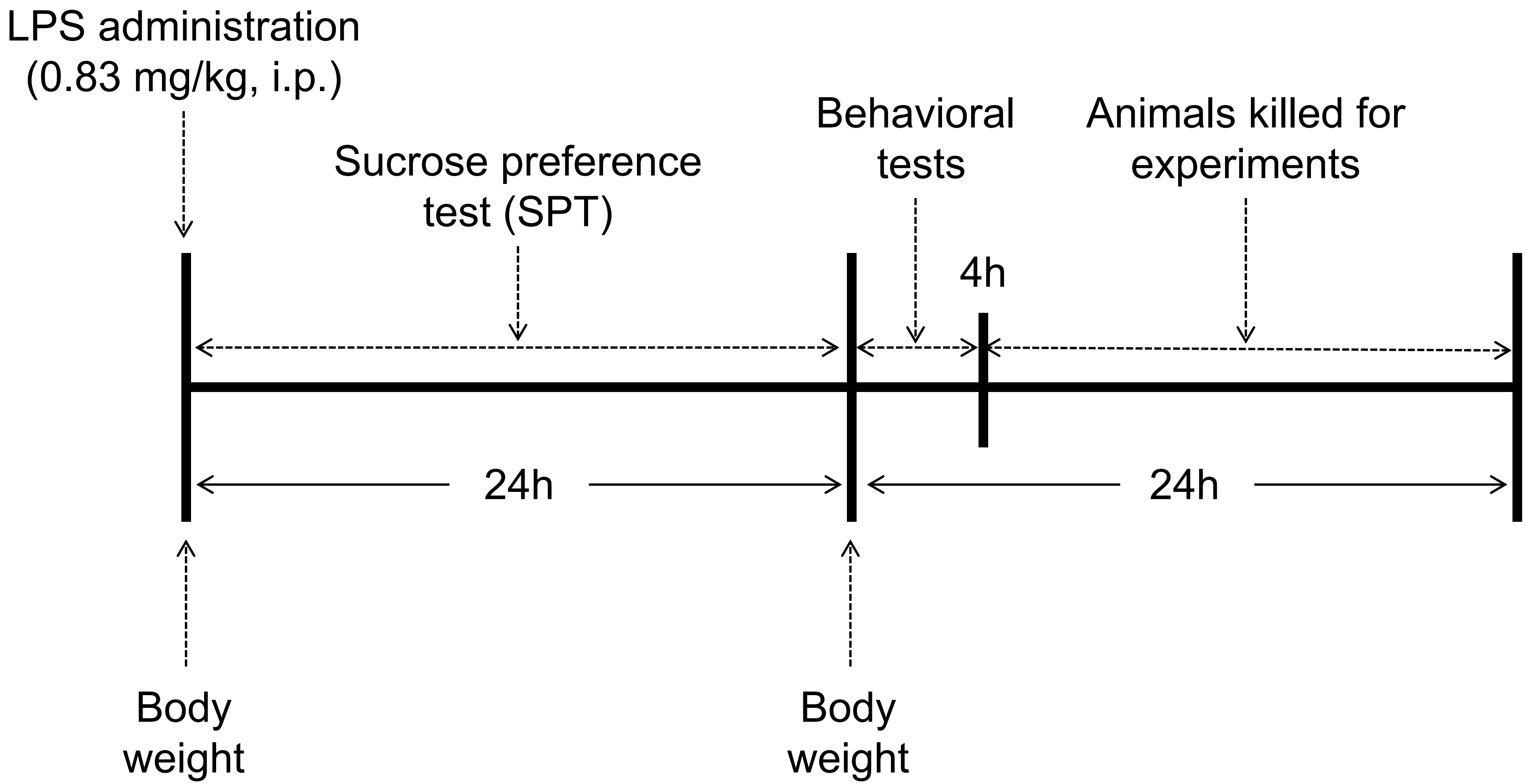
LPS administration
(0.83 mg/kg, i.p.)
Sucrose preference
test (SPT)
Behavioral
tests
Animals killed for
experiments
4h
24h
24h
Body
weight
Body
weight
