# Supplementary figures and images for: Multi‐Omics Analysis Reveals Disturbances of Purine Metabolism and Glutamate Metabolism in the Hippocampus of Lipopolysaccharide‐Induced Mouse Model of Depression
Source: Brain Behav. 2025 May 11;15(5):e70549. doi: 10.1002/brb3.70549 (PMC12066810; doi:10.1002/brb3.70549)

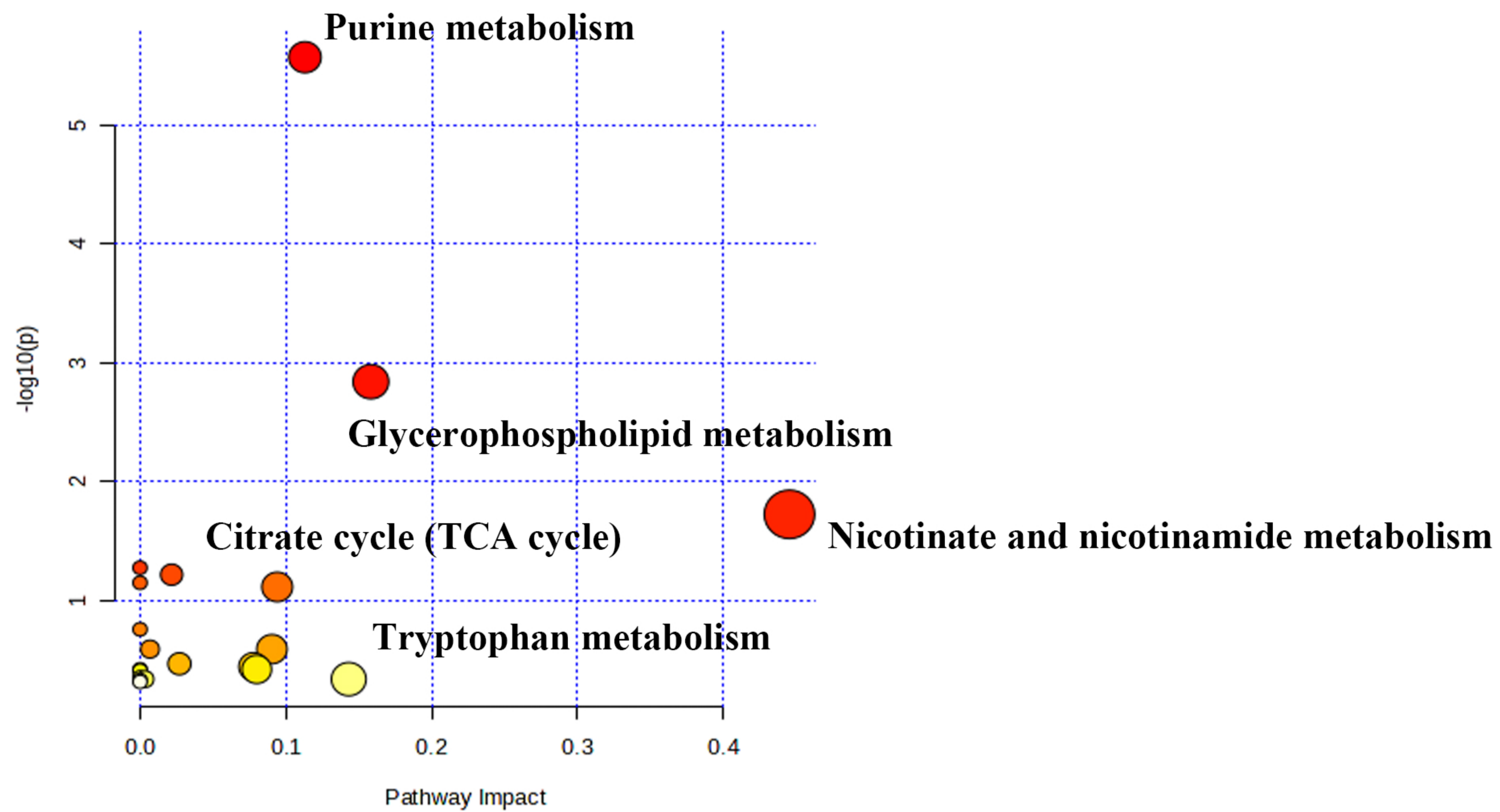

Supplement: Supplementary file 2 — Supporting Information [file BRB3-15-e70549-s001.pdf]
